# Supplementary figures and images for: WGCNA Analysis Revealed the Hub Genes Related to Soil Cadmium Stress in Maize Kernel (Zea mays L.)
Source: Genes (Basel). 2022 Nov 16;13(11):2130. doi: 10.3390/genes13112130 (PMC9690088; doi:10.3390/genes13112130)

A

Pearson correlation between samples

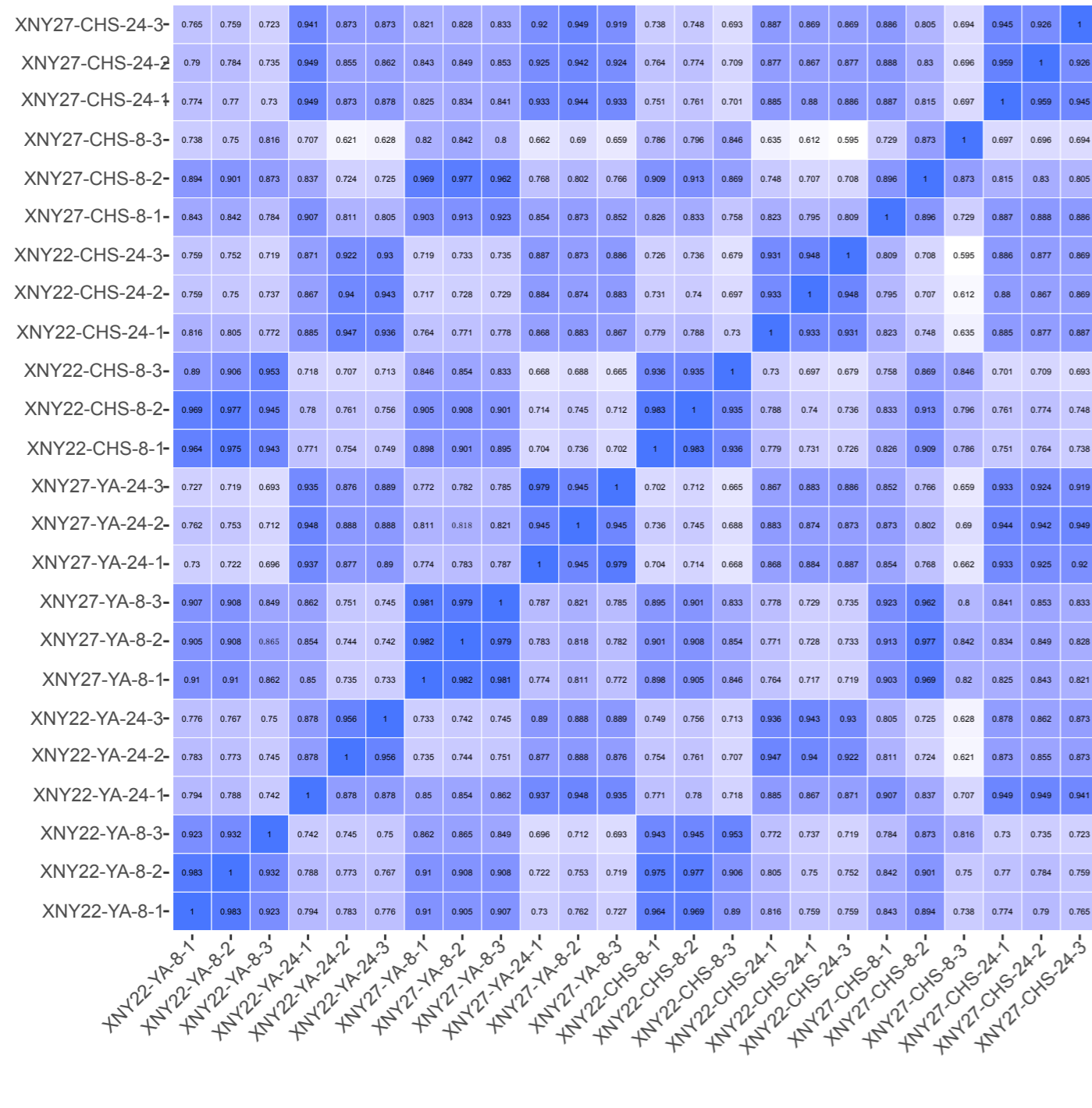

B

PCA

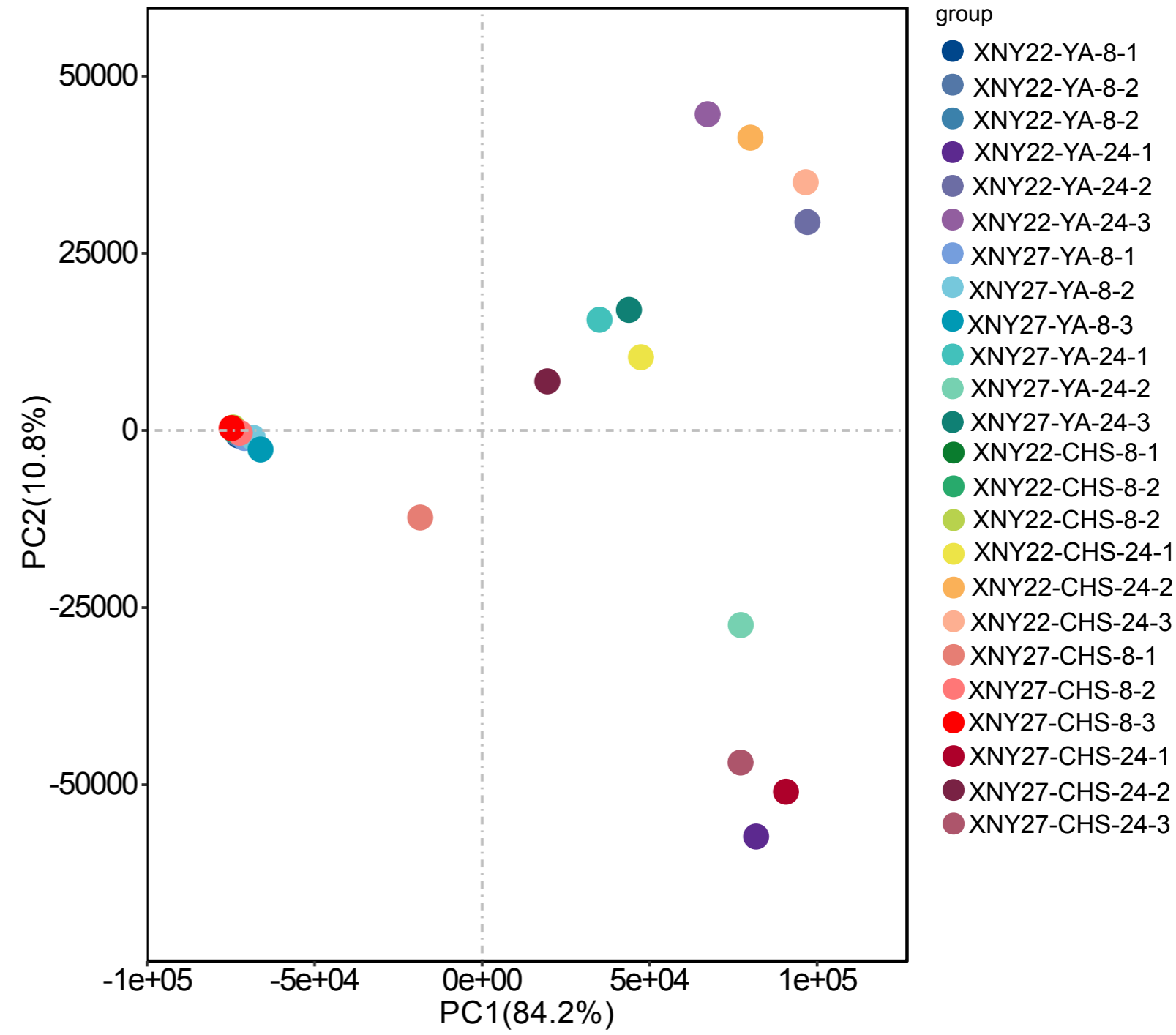

Supplement: Supplementary file 1 [file genes-13-02130-s001.zip › Figure S1.pdf]
